# Teacher autonomy support and externalizing problems: Variations based on growth mindset toward personality and ethnicity

**DOI:** 10.3389/fpsyg.2022.1068751

**Published:** 2022-12-15

**Authors:** Yongfeng Ma, Chunhua Ma, Xiaoyu Lan

**Affiliations:** ^1^College of Educational Science and Technology, Northwest Minzu University, Lanzhou, China; ^2^Promenta Research Center, Department of Psychology, University of Oslo, Oslo, Norway

**Keywords:** externalizing problems, teacher autonomy support, growth mindset, ethnicity, adolescents

## Abstract

**Introduction:**

Given the prevalence of externalizing problems during adolescence, the present study investigated the main and interactive relationships between environmental-level (teacher autonomy support) and person-level (growth mindset toward personality) factors related to externalizing problems. This study further estimated ethnic variations of these relationships among the majority Han and one ethnic minority group (Hui) in China.

**Methods:**

To achieve the research objectives, the study involved 704 Han (*M*_age_ = 12.57; 53.7% female) and 642 Hui adolescents (*M*_age_ = 12.45; 49.4% female) who completed a suite of research questionnaires.

**Results:**

The results of the hierarchical linear regression analysis, after controlling for sociodemographic characteristics and comorbid internalizing problems, showed that teacher autonomy support was directly and negatively related to externalizing problems. This negative relationship was also moderated by growth mindset toward personality and ethnicity. More specifically, a high growth mindset buffered the undesirable effect of low teacher autonomy support on externalizing problems for Hui adolescents but not Han adolescents.

**Discussion:**

The finding from the current research suggests that teacher autonomy support plays a universally beneficial role in youth mental health across two selected ethnic groups. At the same time, identifying the protective role of growth mindset has important practical implications for the design of personalized school-based activities that aim to facilitate adaptive youth behaviors.

## Introduction

Externalizing problems, such as aggression and delinquency, become increasingly evident during adolescence—a developmental period characterized by striking biopsychosocial changes and particularly heightened vulnerabilities (Steinberg and Morris, 2001; Lerner and Castellino, 2002). Past research has consistently shown that externalizing problems, without effective intervention, are associated with several maladaptive functions, such as school underachievement and relationship difficulties (van Lier et al., 2012; Zimmermann et al., 2013; Okano et al., 2020). From a broader perspective, the prevalence of externalizing problems inevitably affects public order and the external environment, leading to economic costs and social distress (Defoe et al., 2013). Empirical research focusing on environmental and personal correlates of externalizing problems in adolescents is therefore essential to gaining evidence-based insights on developing intervention or prevention activities.

Although a large volume of research has already documented the correlates of externalizing problems in adolescence (Lan and Radin, 2020; Zhang and Wang, 2022), several limitations of prior research underscore why further investigation of the topic is warranted. First, at a conceptual level, relatively little research has focused on assessing the direct and joint associations of teacher support based on autonomous motivation (behaving with a sense of volition) and growth mindset toward personality (believing that personality is cultivated through effort) with externalizing problems. Addressing this conceptual gap has profound social and policy implications due to the malleability of both autonomy-supportive teaching and growth mindset toward personality (Yeager and Dweck, 2012; Reeve and Cheon, 2021). Second, past research has long suffered from a narrow focus on a single cultural context (Amir and McAuliffe, 2020), and limited research has explicitly investigated the role of culture/ethnicity in study associations. These gaps in the scholarship are conspicuous considering that several countries have diverse cultural or multi-ethnic backgrounds. Narrowing the gaps would pave the way for a greater conceptual understanding of the universality of autonomy support and growth mindset theories. Practically, documenting the previously identified associations in distinct cultural/ethnic backgrounds would produce novel insights on designing culturally sensitive, school-based activities.

Attempting to fill these knowledge gaps, we follow the socio-ecological theory to investigate the environmental and personal correlates of externalizing problems (Bronfenbrenner and Morris, 2006). This framework emphasizes the joint interplay of environmental-and person-level factors in explaining the variance of adaptive and disruptive behaviors, a framework that has been successfully and widely applied to document the correlates of externalizing problems (Van Heel et al., 2019; Feng and Lan, 2020). Leveraging this informative framework, the current study aimed to extend both the *depth* and *breadth* of prior research by bridging the direct and joint relationships between teacher autonomy support (TAS) and growth mindset toward personality with externalizing problems, and by investigating the ethnic variations of these associations among the majority Han and one ethnic minority group (Hui) in China.

### Teacher autonomy support

Autonomy, as defined by self-determination theory, refers to individuals’ basic psychological need to act on their own volition and to feel psychologically free to behave based on self-endorsed perspectives (Ryan and Deci, 2019). The development of self-awareness and increasing independence highlight the importance of studying autonomy during adolescence (Steinberg and Morris, 2001). Grounded in self-determination theory, significant agents in adolescent social spheres (e.g., teachers) should adopt the student-based attitude, enact autonomy-based instructional behaviors, relate students’ interests and preferences to school activities, and facilitate students’ intrinsic motivational resources and competence (Núñez and León, 2015; Ryan and Deci, 2019; Reeve and Cheon, 2021). Teachers’ emphasis on autonomy support is particularly meaningful to school-aged adolescents as they spend an increasing amount of time at school, which allows teachers to closely supervise the students (Lei et al., 2016). According to empirical studies, TAS has been shown to be negatively related to externalizing problems (Vansteenkiste et al., 2012; Matte-Gagné et al., 2015). These empirical findings, in accordance with the core tenets of self-determination theory (Ryan and Deci, 2019), indicate that energizing students’ intrinsically motivated behaviors and experiences of integration and freedom is vitally important to students’ adaptive behavioral patterns. Despite this converging evidence, an ongoing heated debate focuses on the universality of autonomy support across different cultural contexts. Some researchers propose that the favorable role of autonomy support in healthy youth functioning is fundamental in cultural contexts that emphasize individuality and independence. In contrast, a lack of autonomy and restricted personal choice in Eastern cultures, due to their emphasis on conformity and deference, may not lead to adverse outcomes if done by a trusted figure, such as teachers (Markus and Kitayama, 1991; Iyengar and Lepper, 2000). Revisiting whether Western-developed self-determination theory is cross-culturally generalizable and equally beneficial to youth from Eastern cultural contexts is therefore critical to cooling down this heated debate.

In recent years, an emerging body of research based on Eastern cultural contexts has demonstrated the favorable role of TAS in youth adaptive outcomes, such as facilitating better well-being (Lan and Zhang, 2019), less internalizing (Lan and Mastrotheodoros, 2022), and scarcer externalizing problems (Feng and Lan, 2020). Nevertheless, all the studies have predominantly focused on Chinese Han; the universally favorable role of TAS in youth from different ethnic groups has not yet been confirmed. This burgeoning area of research therefore still warrants further empirical investigation using ethnically diverse populations (Amir and McAuliffe, 2020; Nalipay et al., 2020; Haw and King, 2022).

Further, the striking individual differences in the relationships between TAS and externalizing problems are underexplored. Based on the socio-ecological framework (Bronfenbrenner and Morris, 2006), individual characteristics could moderate the relationship between environmental influences and externalizing problems. One important person-level variable to be considered is growth mindset, as elaborated upon below.

### Growth mindset

Growth mindset refers to an implicit belief that personal attributes (e.g., intelligence and personality) are changeable and can be developed and cultivated through practice and efforts (Dweck et al., 1995; Yeager and Dweck, 2012). In recent years, several empirical studies have highlighted the critical function of growth mindset in academic settings, including better self-regulation abilities and academic performance (Burnette et al., 2013; Rattan et al., 2015). Past research, however, has predominantly focused on growth mindset toward intelligence and its functions within only learning-related outcomes. Extending this line of scholarship by centering on growth mindset toward other personal attributes (e.g., personality) and relating how that mindset affects other outcomes beyond academic domains is conceptionally important and practically meaningful. Past research has shown that individuals with great growth mindset not only can continuously invest their efforts even after failure but also can use positive strategies to regulate individual emotions and behaviors (Claro et al., 2016; Han et al., 2018). Intervention studies have also revealed that youth with heightened vulnerabilities particularly benefit more from growth mindset interventions compared to those who are psychologically flourishing (Sisk et al., 2018). Therefore, growth mindset could buffer the adverse effect of unfavorable social contexts (i.e., low TAS) on externalizing problems.

Further, mounting evidence has shown that the role of growth mindset in a few functional domains is not universal across cultures (Dong and Kang, 2022). For instance, youth from Eastern cultural contexts are more likely than their peers in Western ones to develop a fixed mindset (Sun et al., 2021; Dong and Kang, 2022). The authors of such research suggested that parents from Eastern societies often show excessive concerns regarding children’s academic performance due to the cultural emphasis on competitiveness and exam-oriented education, consequently restricting the development of children’s non-cognitive abilities. Instead of examining the universal role of growth mindset, researchers should explore the culture-specific context of growth mindset in the relationship between TAS and externalizing problems. In the present research, we therefore extended prior research by investigating study associations in adolescents from two ethnic groups (i.e., Han and Hui).

### Ethnicity

For this study, we selected two ethnic groups in China: the ethnic majority Han and one ethnic minority group (Hui, one of the largest ethnic minorities in China) since the two groups differ significantly in the social interactions and core beliefs that could significantly influence study associations. Traditionally, Confucianism has predominantly influenced Han’s cultural values, highly emphasizing collectivistic welfare, social harmony, and interdependence (Bond, 2010). In the past decade, however, traditional socio-cultural values have quickly evolved due to rapid economic growth and subsequent fierce competition (Chen et al., 2010). Living in such an economically developed society could promote individualism, allowing individuals to prioritize personal goals and freedoms (Santos et al., 2017). From this perspective, social relationships (e.g., teacher-student relationships) are somewhat being reshaped; personal choice and individual competition are more underscored in contemporary Han society.

In contrast, the Islamic religion plays an essential role in socio-cultural values and lifestyles among the Hui (Chuah, 2004). Although the Hui in contemporary society are acculturated by speaking in Mandarin and adopting modern clothing styles like the Han, the Hui still retain religious affiliation and the associated customs and dietary practices (Liu et al., 2012). For instance, cultivating children’s etiquette and teaching them to respect and obey social order and rules are critical steps in the Hui culture. Despite the aforementioned intercultural differences, only a handful of researchers have examined this ethnic group comparison in psychological research (e.g., Ha et al., 2020; Zhang and Ng, 2022). Disentangling the ethnic variations of study associations would further enrich the existing literature and make valuable contributions to culturally sensitive intervention or prevention activities that aim to facilitate adolescents’ adaptive behavioral patterns.

### The present study

Given the prevalence of externalizing problems during adolescence, the present study leveraged the socio-ecological framework to investigate its environmental (TAS) and individual (growth mindset) correlates. More importantly, this study aimed to examine the universality of the results by testing the associations among adolescents from two ethnic groups. Specifically, the following research questions (RQ) were addressed:

RQ1: Is high TAS related to low externalizing problems in adolescents?

Based on self-determination theory (Ryan and Deci, 2019) and earlier empirical studies (Lan and Zhang, 2019; Feng and Lan, 2020), we expected that TAS would be negatively related to externalizing problems (Hypothesis 1; main effect).

RQ2: Does the expected negative association between TAS and externalizing problems vary (moderated) by growth mindset and/or two ethnic groups?

**Figure 1:**
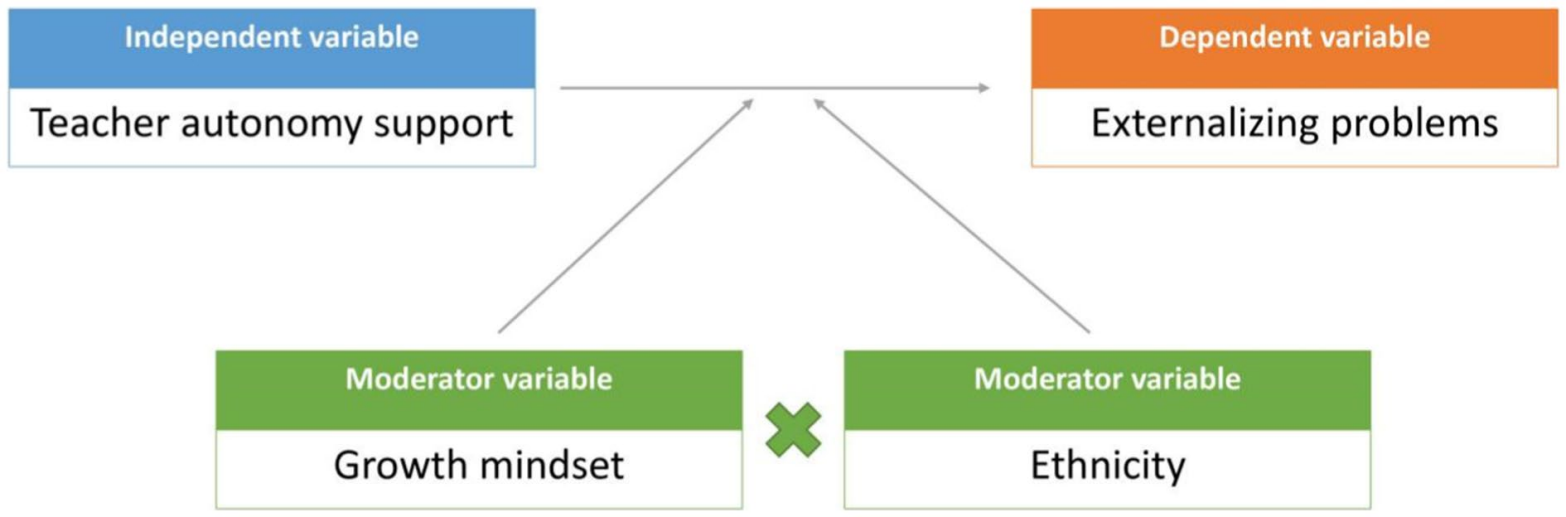
Conceptual model with two moderators. Age, gender, family socioeconomic status, and internalizing problems were regarded as covariates.

Based on prior research (Sisk et al., 2018; Niu et al., 2020), we hypothesized that high growth mindset might buffer the negative effect of low TAS on externalizing problems (Hypothesis 2a; two-way interaction). Further, based on the intercultural differences, we expected that the moderating role of growth mindset might be more heightened in Han adolescents than in Hui adolescents (Hypothesis 2b; three-way interaction). A conceptual model is presented in Figure 1.

When addressing the two RQs, we statistically controlled for age, gender, and family socioeconomic status to preclude sociodemographic confounding effects and robustly examine the research hypotheses. We also controlled for the levels of internalizing problems to adjust for possible comorbidity with externalizing problems (Achenbach et al., 2016).

## Materials and methods

### Participants and procedure

Prior to data collection, the institutional review board of the Northwest Minzu University approved this project. Informed consent was obtained from both parents and participants. Participants’ rights, confidentiality, and anonymity were highlighted as our priorities when conducting this investigation. Our research project was based on school collaborations and received immense support from the local Education Bureau and school authorities, ensuring a high participation rate (approximately 90%). Such collaboration was established on an annual basis in a few public primary and secondary schools in Lin-Xia, located in northwest China. Lin-Xia is a county-level city that serves as one autonomous prefecture for the Hui people, and thus gathering research data from this location is suitable to address current RQs. Our research group members, together with head teachers in each classroom, administrated this survey during school hours using a packet of well-design questionnaires written in simplified Chinese.

The final sample comprised 1,346 (51.6% girls) adolescents aged between 10 and 15, with an average age of 12.51. Among them, 704 was self-identified as Han (*M_age_* = 12.57; *SD* = 1.44; 54.1% female) and 642 was Hui (*M_age_* = 12.45; *SD* = 1.37; 50.1% female). During data collection, they attended the same schools from fourth to eighth grades. These two ethnic groups of adolescents came from the same classrooms, and the collaboration school did not administrate the streaming practice. Hence, their sociodemographic features were comparable (see Table 1).

**Table 1:** Sociodemographic characteristics.

	Han (*n* = 704)	Hui (*n* = 642)
**Age (***M*** ± ***SD* **years**)	12.57 ± 1.44	12.45 ± 1.37
**Gender (% girls)**	53.7%	49.4%
**Parental education (father/mother)**		
Undergraduate education or higher	204 (29.0%)/179 (25.4%)	146 (22.7%)/122 (19.0%)
High school	225 (32.0%)/205 (29.1%)	177 (27.6%)/139 (21.6%)
Secondary school or lower	188 (26.6%)/226 (32.1%)	219 (34.1%)/265 (41.4%)
Missing values	87 (12.4%)/94 (13.4%)	100 (15.6%)/116 (18.1%)
**Family socioeconomic status**		
Low-income families	68 (9.6%)	56 (8.8%)
Medium-income families	325 (46.2%)	302 (47.0%)
High-income families	311 (44.2%)	284 (44.2%)

### Measures

#### Externalizing problems

Adolescents reported their externalizing problems using the subscales (i.e., conduct problems and hyperactivity) of the Strengths and Difficulties Questionnaire (SDQ; Goodman, 1997). Adolescents indicated how they felt or behaved over the past 6 months on 10 items. One item example is “I get very angry and often lose the temper.” Ratings were obtained on a three-point scale ranging from (*0-not true*) to (**2-*certainly true*). Sum scores were calculated, and higher scores were indicative of higher levels of externalizing problems. Prior research has exhibited good reliability and validity of the SDQ in Chinese adolescents (Lan et al., 2020). In the present samples, Cronbach’s alpha was adequate for adolescents from two ethnic groups (Cronbach’s alpha = 0.63 and McDonald’s omega = 0.65 for Han adolescents; Cronbach’s alpha = 0.62 and McDonald’s omega = 0.63 for Hui adolescents).

#### Teacher autonomy support

Adolescents rated autonomy support provided by their teachers using the shortened version of the students’ perceived autonomy-supportive teaching questionnaire (Williams and Deci, 1996). Youth reported on these six items (e.g., my head teacher tries to understand how I see things before suggesting a new way to do things), and response options range from *strongly disagree* (1) to *strongly agree* (7). The mean score was calculated, and higher values denoted greater autonomy-supportive practices provided by head teachers in each classroom. Good internal consistency has been exhibited in prior research on Chinese youth (Lan and Zhang, 2019). The internal consistency for the measure of TAS was good in the present samples (Cronbach’s alpha = 0.89 and McDonald’s omega = 0.89 for Han adolescents; Cronbach’s alpha = 0.91 and McDonald’s omega = 0.91 for Hui adolescents).

#### Growth mindset

Growth mindset was assessed with the Implicit Theory Scale (Dweck et al., 1995). Of this scale, three items were indicators of growth mindset toward personality (e.g., People can do things differently, but the important parts of who they are cannot really be changed.) Response options range from 1 (*strongly disagree*) to 6 (*strongly agree*). We calculated the average score of all the reverse-coded items, and higher scores were indicative of a stronger growth mindset toward personality. Good internal consistency has been exhibited in prior research on Chinese youth (Ma et al., 2022). In the current samples, the internal consistency of this scale was acceptable (Cronbach’s alpha = 0.71 and McDonald’s omega = 0.71 for Han adolescents; Cronbach’s alpha = 0.66 and McDonald’s omega = 0.66 for Hui adolescents).

#### Covariates

Sociodemographic information, including age, gender, and family socioeconomic status, was measured at the beginning of this investigation. More specifically, we assessed family socioeconomic status using the four-item family affluence scale (Boyce et al., 2006) because conventional indicators (e.g., parental education and occupation) often result in a large number of missing values. Further, we used the SDQ (Goodman, 1997) to assess internalizing problems to better isolate the relations between variables of interest and externalizing problems. The internal consistency of this scale was acceptable in the present samples (Cronbach’s alpha = 0.68 and McDonald’s omega = 0.70 for Han adolescents; Cronbach’s alpha = 0.67 and McDonald’s omega = 0.69 for Hui adolescents).

### Data analysis

The analytical plan was divided into three sections: (a) preliminary analysis, (b) descriptive analysis, and (c) focal analysis to address the RQs investigated. All data analyses were conducted using SPSS 27.0 (IBM Corp, 2020). In terms of preliminary analysis, we first calculated the intraclass correlation coefficient (ICC), regarding the classroom as a cluster and externalizing problem as the outcome, given that the data set analyzed had a nested design (i.e., students were nested in 56 classrooms). The results showed low variability (ICC < 0.05) of externalizing problems across different classrooms, we therefore did not conduct a mixed model in the further course of the analysis. Data were subsequently screened for multivariate normality and outliers (Ma et al., 2020), and 11 cases were omitted from this step, resulting in the final sample size reported above.

In terms of descriptive analysis, the means, standard deviations, and bivariate correlations among study variables, separated by the ethnic groups, are presented. Additionally, independent *t*-tests/Chi-square tests, where applicable, were conducted to estimate ethnic group differences in study variables.

Regarding the focal analysis, we applied hierarchical linear modeling to document the direct and joint (moderation) relations of TAS, growth mindset, and ethnicity with externalizing problems (Lindenberger and Pötter, 1998). The analysis was conducted in four steps in a sequential manner. In the first step, only confounding variables were entered. In the second step, the main effects were assessed by directly putting TAS, growth mindset, and ethnicity inside the model. In the third step, the two-way multiplicative interactions (TAS X growth mindset, TAS X ethnicity, and growth mindset X ethnicity) were additionally entered, and in the final step, the three-way interaction term among them (TAS X growth mindset X ethnicity) were additionally created. During this process of the analysis, categorical variables were dummy-coded, and continuous variables were centered around their means prior to entry into the model to reduce multicollinearity (Robinson and Schumacker, 2009). The initial inspection of the percentage of the missing values per each focal variable was less than 5%, and Full information maximum likelihood estimator (FIML) was therefore implemented to handle missing data (Enders, 2001). In addition, significant interactions were probed using the visualized interaction graphs, simple slope analyses, and the Johnson-Neyman technique (Aiken and West, 1991; Hayes and Matthes, 2009). In all these analyses, the level of significance was interpreted at *p* < 0.05, and the 95% confidence intervals should not contain zero.

## Results

### Descriptive analyses

Table 2, separated by two ethnic groups, contains bivariate correlations among study variables, along with the means and standard deviations. As shown in Table 2, in both ethnic groups of adolescents, TAS was negatively related to externalizing problems, initially supporting the first hypothesis. Independent *t*-tests/Chi-square tests revealed no statistically significant differences across the two ethnic groups in terms of study variables and covariates, confirming that their sociodemographic characteristics were comparable.

**Table 2:** Descriptive statistics and bivariate correlations.

	Han (*n* = 704)		Hui (*n* = 642)				*t*/χ^2^	1	2	3	4	5	6	7
	*M*	*SD*	Range	*M*	*SD*	Range
1. Externalizing problems	6.97	3.18	0–17	7.24	3.29	0–17	−1.57	—	−0.26^***^	−0.06	−0.03	−0.06	−0.02	0.54^***^
2. Teacher autonomy support	4.94	1.20	1–7	4.79	1.46	1–7	1.94	−0.26^***^	—	−0.05	0.18^***^	0.06	0.04	−0.23^***^
3. Growth mindset	3.76	1.15	1–6	3.83	1.15	1–6	0.66	−0.11^**^	0.05	—	0.07	0.01	0.02	−0.06
4. Age	12.57	1.44	10–15	12.45	1.37	10–15	1.47	−0.07	0.20^***^	0.15^***^	—	0.09^*^	−0.08^*^	−0.07
5. Gender^a^	-	-	1–2	-	-	1–2	2.21	−0.16^***^	0.09^*^	0.01	0.02	—	0.01	−0.01
6. Family SES	5.01	1.74	0–9	5.07	1.81	0–9	−0.66	−0.02	0.12^**^	−0.05	−0.08^*^	−0.02	—	−0.06
7. Internalizing problems	6.45	3.43	0–18	6.82	3.57	0–18	−1.92	0.54^***^	−0.15^***^	−0.13^***^	−0.10^**^	−0.04	−0.03	—

*N* = 1,346. ^a^coded as 1 = male, 2 = female. SES, socioeconomic status. Correlation coefficients displayed below the diagonal are for Han adolescents, above for Hui adolescents. ^*^p < 0.05, ^**^p < 0.01, and ^***^p < 0.001.

### Focal analyses

Table 3 presents the results of the hierarchical linear regression that included main effects, two-and three-way interactions. In the first step, as shown in Table 3, the confounding variables explained 30.3% variance of externalizing problems. Specifically, boys reported higher externalizing problems than girls, and internalizing problems were significantly and positively correlated with externalizing problems. Nevertheless, age and family socioeconomic status were not significantly related to externalizing problems. In the second step, focal variables additionally explained 2.5% variance of externalizing problems. Both TAS and growth mindset were negatively related to externalizing problems, whereas ethnicity was not. The first hypothesis was supported. In the third step, three two-way interactions among focal variables additionally explained 0.4% variance of externalizing problems. Of these two-way interaction terms, the interaction between TAS and growth mindset was significantly related to externalizing problems, whereas the remaining two interaction terms were not significant. In the final step, the significant three-way interaction additionally explained 0.2% variance of externalizing problems, and this term was significantly related to externalizing problems.

**Table 3:** Hierarchical regression model predicting externalizing problems.

	*b*	*b SE*	*t*	*p*	*R* ^2^	△*R*^2^	△*F*
**Step 1**							
Age	−0.00	0.05	−0.00	0.996			
Gender^a^	−0.63	0.15	−4.26	< 0.001			
Family socioeconomic status	0.00	0.04	0.06	0.953			
Internalizing problems	0.50	0.02	23.37	< 0.001	0.303	0.303	143.8^***^
**Step 2**							
Age	0.08	0.05	1.45	0.146			
Gender	−0.57	0.15	−3.86	< 0.001			
Family socioeconomic status	0.03	0.04	0.65	0.516			
Internalizing problems	0.47	0.02	21.90	< 0.001			
Teacher autonomy support	−0.39	0.06	−6.79	< 0.001			
Growth mindset	−0.13	0.06	−1.97	0.049			
Ethnicity^b^	0.03	0.15	0.19	0.846	0.328	0.025	16.39^***^
**Step 3**							
Age	0.07	0.05	1.40	0.160			
Gender	−0.56	0.15	−3.85	< 0.001			
Family socioeconomic status	0.03	0.04	0.70	0.481			
Internalizing problems	0.47	0.02	22.04	< 0.001			
Teacher autonomy support	−0.92	0.19	−4.95	< 0.001			
Growth mindset	−0.71	0.24	−2.97	0.003			
Ethnicity	−0.77	0.74	−1.05	0.296			
Teacher autonomy support × Growth mindset	0.12	0.04	2.66	0.008			
Teacher autonomy support × Ethnicity	0.14	0.11	1.25	0.211			
Growth mindset × Ethnicity	0.04	0.13	0.28	0.779	0.332	0.004	3.06^*^
**Step 4**							
Age	0.08	0.05	1.41	0.158			
Gender	−0.58	0.15	−3.93	< 0.001			
Family socioeconomic status	0.03	0.04	0.73	0.465			
Internalizing problems	0.47	0.02	21.94	< 0.001			
Teacher autonomy support	−0.53	0.27	−1.96	0.050			
Growth mindset	−0.18	0.35	−0.52	0.604			
Ethnicity	2.70	1.86	1.45	0.148			
Teacher autonomy support × Growth mindset	0.01	0.07	0.19	0.850			
Teacher autonomy support × Ethnicity	−0.56	0.36	−1.54	0.123			
Growth mindset × Ethnicity	−0.86	0.46	−1.87	0.062			
Teacher autonomy support × Growth mindset × Ethnicity	0.18	0.09	2.03	0.043	0.334	0.002	4.11^*^

*N* = 1,346. ^a^Coded as 1 = male, 2 = female; ^b^Coded as 1 = Han adolescents, 2 = Hui adolescents; ^*^p < 0.05, ^***^p < 0.001.

**Figure 2:**
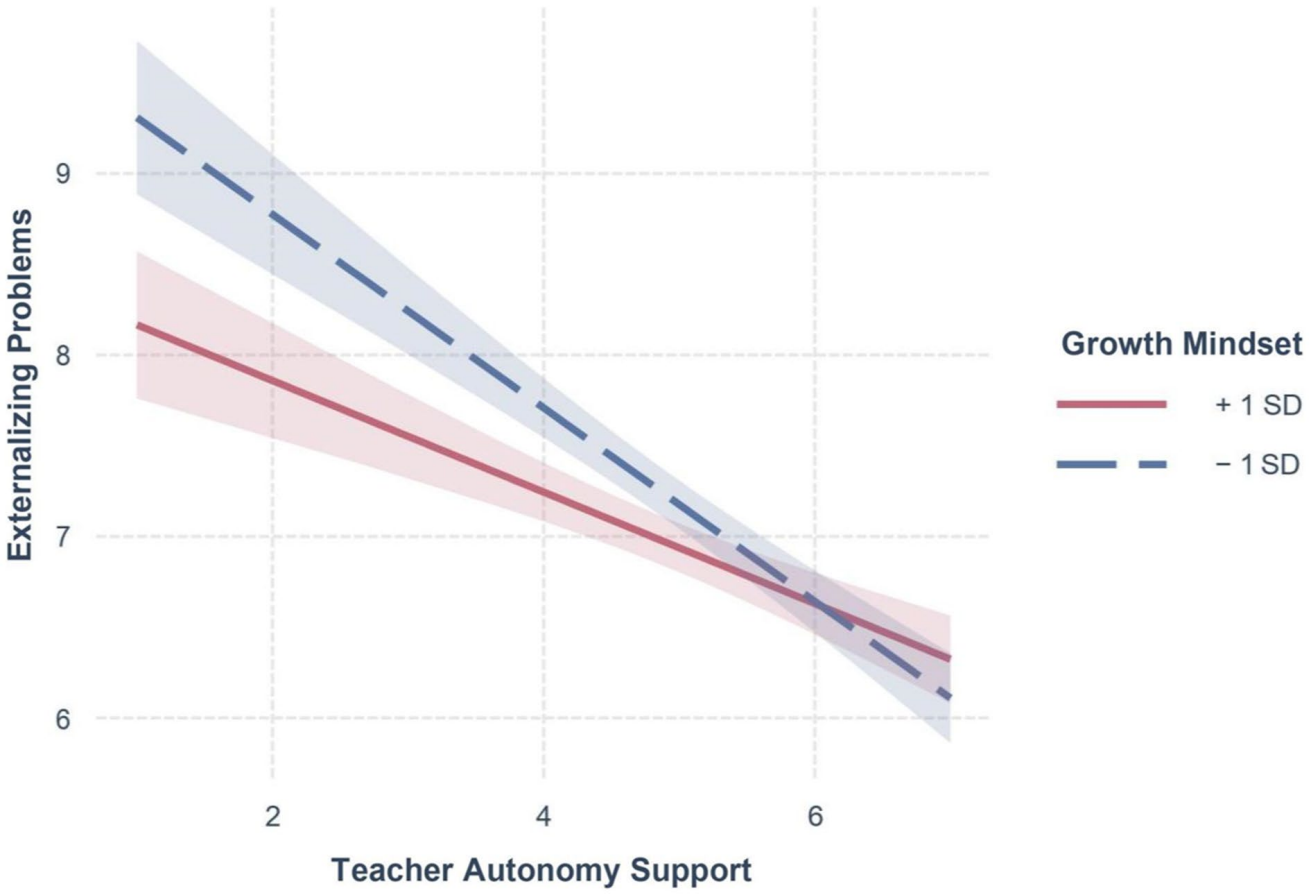
Relation between teacher autonomy support and externalizing problems as a function of growth mindset. *N* = 1,346. Broad bands in the figure indicate 95% CIs. Low growth mindset represents 1 *SD* below the mean, whereas high growth mindset represents 1 *SD* above the mean.

Figure 2 visualizes the interactive patterns between TAS and growth mindset on externalizing problems. Specifically, the relation between TAS and externalizing problems remained significantly negative at both high (*b* = −0.29, *SE* = 0.07, 95% CI [−0.44, −0.15], *t* = −3.99, *p* < 0.001), and low growth mindset (*b* = −0.53, *SE* = 0.07, 95% CI [−0.69, −0.38], *t* = −6.77, *p* < 0.001). From a descriptive point of view, high growth mindset significantly buffered against the negative effect of low TAS on externalizing problems, supporting the second hypothesis (2a).

**Figure 3:**
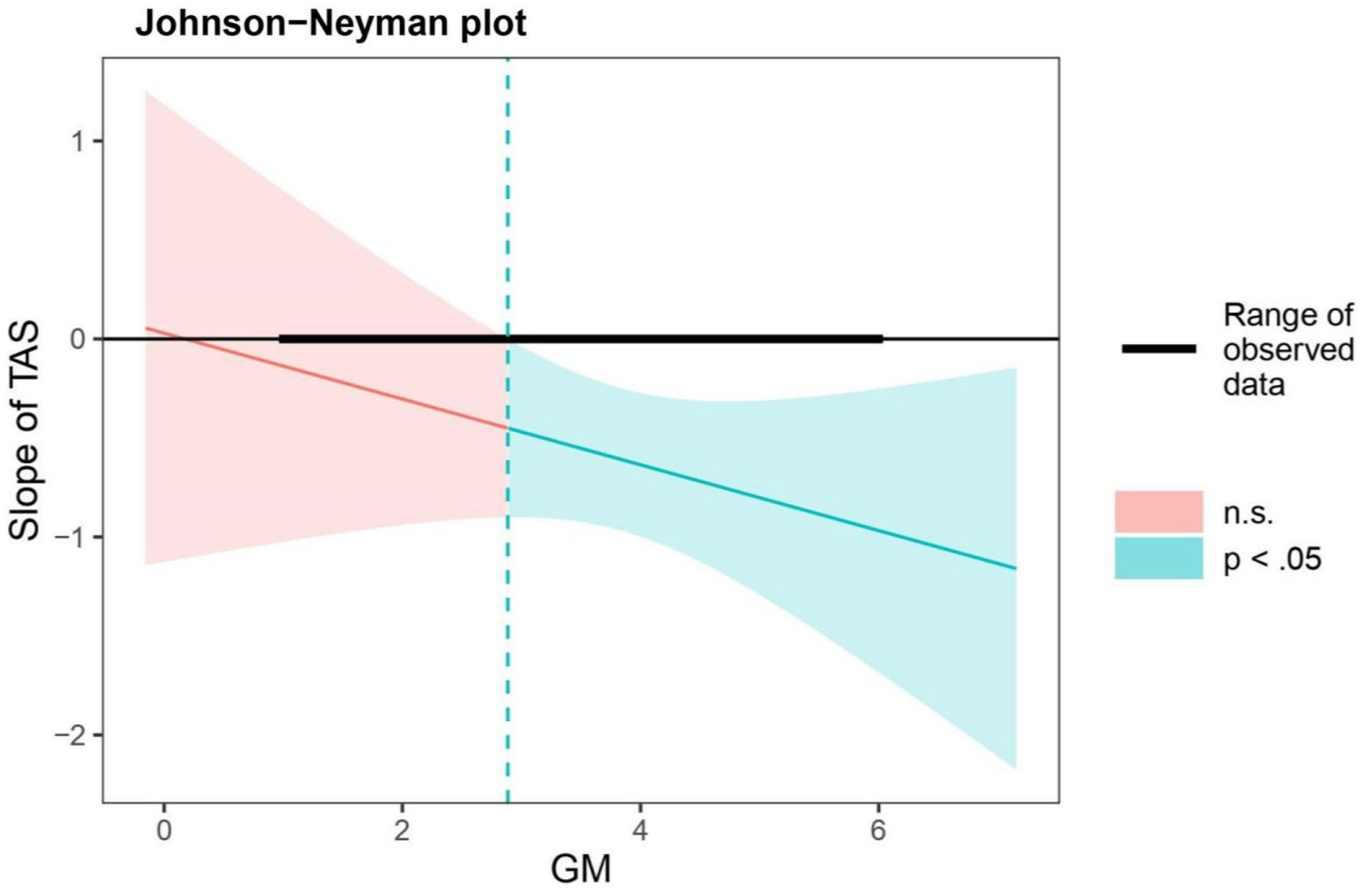
Johnson-Neyman regions of significance and confidence bands for the conditional relation between teacher autonomy support and externalizing problems as a function of growth mindset. *N* = 1,346. TAS, teacher autonomy support; and GM, growth mindset.

In terms of significant regions of growth mindset, the results of the Johnson-Neyman technique showed that, when the scores of growth mindset exceeded 2.89, the relation between TAS and externalizing problems was significantly negative (see Figure 3).

**Figure 4:**
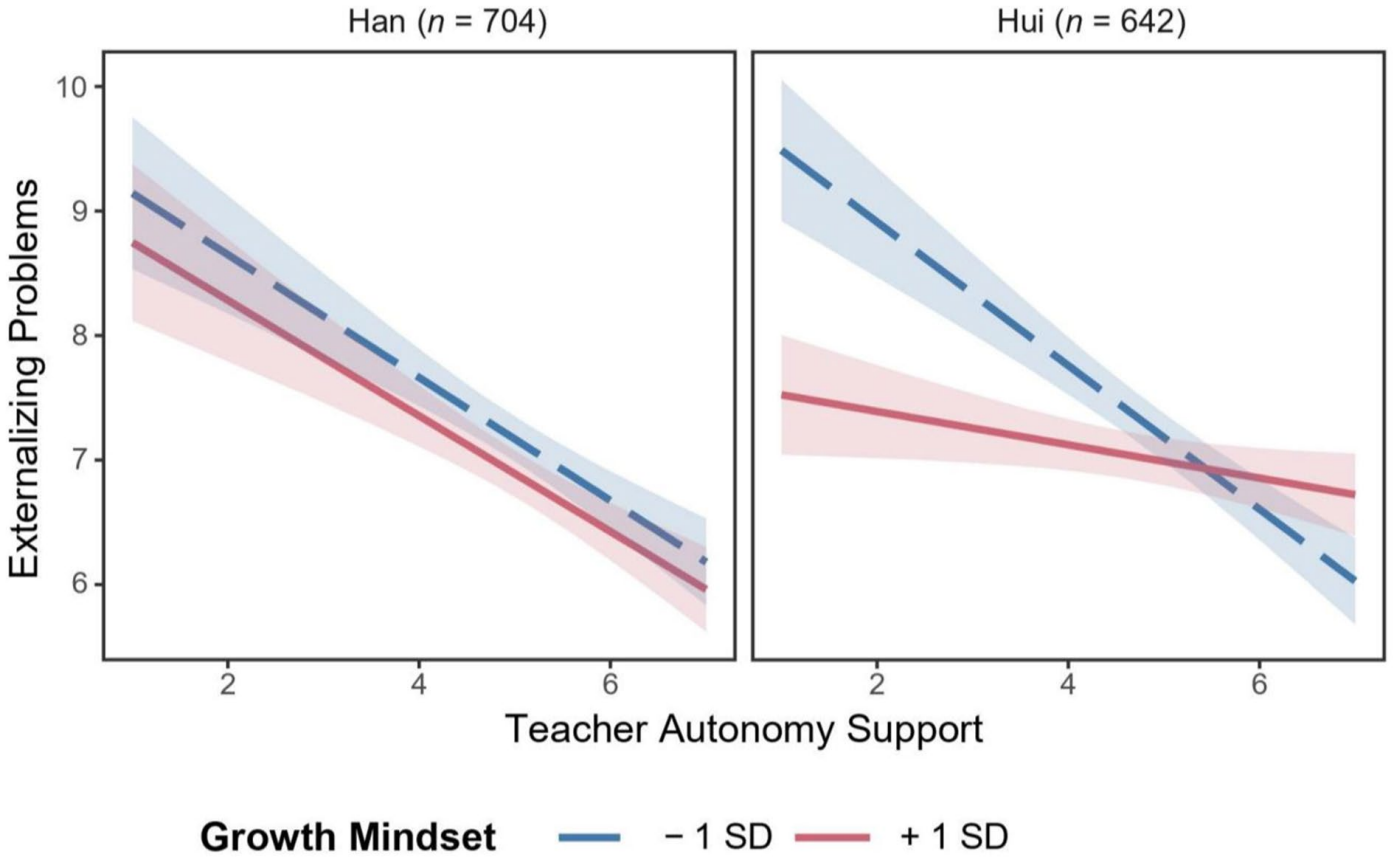
Relation between teacher autonomy support and externalizing problems as a function of growth mindset and ethnicity. *N* = 1,346. Broad bands in the figure indicate 95% CIs. Low growth mindset represents 1 *SD* below the mean, whereas high growth mindset represents 1 *SD* above the mean.

Figure 4 visualizes the three-way interaction. Specifically, for Han adolescents, the association between TAS and externalizing problems was significantly negative at both high (*b* = −0.49, *SE* = 0.11, 95% CI [−0.71, −0.26], *t* = −4.30, *p* < 0.001), and low growth mindset (*b* = −0.46, *SE* = 0.11, 95% CI [−0.69, −0.23], *t* = −4.03, *p* < 0.001). In contrast, for Hui adolescents, the negative association between TAS and externalizing problems was only significant at low growth mindset (*b* = −0.57, *SE* = 0.10, 95% CI [−0.78, −0.36], *t* = −5.39, *p* < 0.001), but not at high growth mindset (*b* = −0.13, *SE* = 0.09, 95% CI [−0.31, 0.04], *t* = −1.44, *p* = 0.14; see Figure 4). From a descriptive point of view, for Han adolescents, the negative association between TAS and externalizing problems was independent of growth mindset levels. In contrast, for Hui adolescents, high growth mindset significantly buffered against the negative effect of low TAS on externalizing problems.

**Figure 5:**
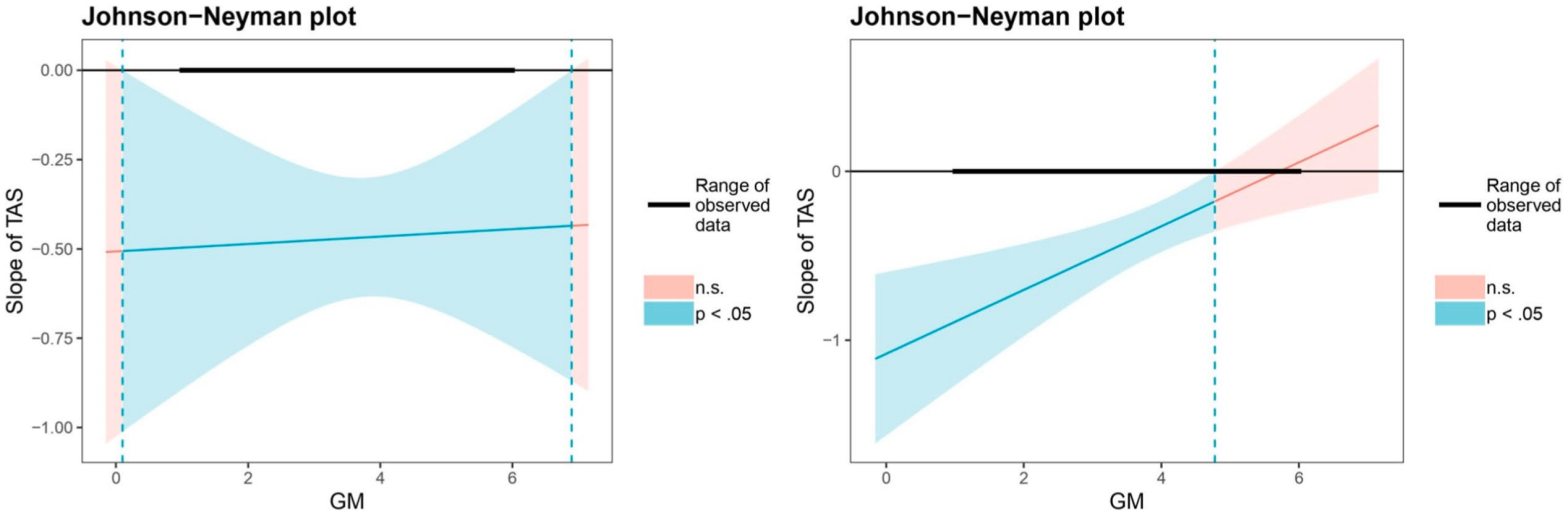
Johnson-Neyman regions of significance and confidence bands for the conditional relation between teacher autonomy support and externalizing problems as a function of growth mindset and ethnicity (Han adolescents—left panel; Hui adolescents—right panel). *N* = 1,346. TAS, teacher autonomy support and GM, growth mindset.

In terms of significant regions of moderators, the association between TAS and externalizing problems was significant for Han adolescents in all levels of growth mindset; by contrast, when the scores of growth mindset exceeded 4.78, this association was not significant for Hui adolescents (see Figure 5).

## Discussion

Leveraging the socio-ecological framework, the present research aimed to examine the direct and interactive associations of TAS and growth mindset toward personality with externalizing problems among adolescents. This study also estimated the cross-cultural universality of these associations by examining the moderating role of ethnicity therein. Below, we discuss how these aims were met *via* a discussion of the findings.

In accordance with the first hypothesis (main effect), the current findings showed that TAS was negatively related to externalizing problems. This pattern further substantiates the self-determination theory (Ryan and Deci, 2019) and corroborates extant research derived from Western cultural contexts (Vansteenkiste et al., 2012), demonstrating that TAS is vital when adjusting youths’ disruptive behaviors in an Eastern cultural context. One possible interpretation is that personal choice and autonomy have been increasingly emphasized in school contexts due to rapid economic growth and the subsequent tremendous socio-cultural adjustment. Cultivating autonomy, such as encouraging students to express personal opinions and cultivating students’ self-awareness (Yu et al., 2016), helps equip students with better capacities to deal with possible difficulties triggered by fierce social competition. Additionally, during adolescence, the amount of time spent at school increases compared to earlier ages, and teachers often act as key figures in fostering youth adaptive behavioral patterns (Pianta, 1999). In this context, teachers can recognize adolescents’ externalizing problems in a timely manner and provide subsequent guidance (Reeve, 2002; Reeve and Cheon, 2021). TAS can facilitate youth competencies by stimulating adolescent intrinsic motivation and inducing adaptive behaviors rather than putting external pressure on adolescents, which then effectively regulates their behaviors and deters them from engaging in problem behaviors.

In line with the second hypothesis (Hypothesis 2a), the results generated from the two-way interaction showed that high growth mindset counteracted the detrimental effect of low TAS on externalizing problems. This finding also aligns with prior research (Burnette et al., 2013; Claro et al., 2016; Sisk et al., 2018), highlighting the protective role of growth mindset under heightened vulnerabilities. Adolescents with a high growth mindset toward personality, under unfavorable contexts (low TAS), may attempt to hold favorable beliefs and reappraise the situation from a positive and adaptive perspective, and still exhibit adaptive behavior patterns and report fewer externalizing problems.

Further, three-way interaction among study variables (Hypothesis 2b) was confirmed, although the interactive patterns were not expected. Specifically, the buffering role of growth mindset was highlighted for Hui adolescents but not for Han adolescents. The findings, in concert with prior literature, highlight the heterogeneity in the association between growth mindset and relevant outcomes across cultures (Sun et al., 2021; Dong and Kang, 2022). Islamic religion particularly restrains problem behaviors and emphasizes tenacity in response to stress and failure (Chuah, 2004). Under this religious influence, Hui adolescents may hold positive beliefs that unfavorable contexts, such as low TAS, are situational and can be improved upon. The buffering role of growth mindset in Hui adolescents is hence more pronounced than in Han adolescents. At the same time, Han adolescents are exposed to highly competitive environments, and parental overemphasis on diligence and academic outcomes may let such adolescents adopt inflexible learning and coping strategies, consequently eroding adolescents’ growth mindset.

Along with the significant findings outlined above, a few limitations should be acknowledged. First, the current study relied on self-reported measurements; although they are methodologically sound, such measurements are subject to common method variance and social desirability (Podsakoff et al., 2003). Adolescents’ externalizing behaviors, for instance, may be better observed and reported by other informants, including teachers and parents. Second, notably, the data set collected was built *via* a convenience sampling method, and adolescents were recruited from only Northwest China. Given that Han and Hui populations are highly distributed across the country, this limitation may restrain the generalizability of the findings.

These documented limitations notwithstanding, the current study has important theoretical and practical implications. Theoretically, the current study enriches self-determination and mindset theories, contributing to the discussion of autonomous motivation and growth mindset as universal or cultural phenomena. This study overall also offers broad support for the cross-cultural generalization of self-determination theory, indicating that the beneficial role of autonomy support should be consistent across different cultural/ethnic groups. Interactive patterns discovered between environmental and individual factors help enrich the socio-ecological framework as well, comprehensively elucidating the developmental processes of youth externalizing problems. More importantly, the current study generates important practical implications for culturally sensitive intervention or prevention programs that aim to mitigate adolescents’ externalizing problems. Teachers from collectivistic cultural contexts, for instance, should adopt an autonomy-supportive rather than a controlling style in contemporary societies, which is conducive to youth adaptive behavioral patterns. Further, while growth mindset serves as a protective factor that buffers the detrimental effect of low TAS on youth adaptive behavioral patterns, the specific ethnic/cultural background should still be carefully considered in this context.

## Data availability statement

The raw data supporting the conclusions of this article will be made available by the authors, without undue reservation.

## Ethics statement

The studies involving human participants were reviewed and approved by Northwest Minzu University. Written informed consent to participate in this study was provided by the participants’ legal guardian/next of kin.

## Author contributions

YM and CM conceived and drafted the manuscript. XL performed the statistical analyses and critically revised the manuscript. All authors contributed to the article and approved the submitted version.

## Funding

This study was supported by Research on Northwest Adolescent Education and Development (Northwest Minzu University, Project ID. 1110130135) and the Education and Teaching Reform Research Project of Northwest Minzu University (Nos. 2021XJYBJG-32 and YLKC-13).

## Conflict of interest

The authors declare that the research was conducted in the absence of any commercial or financial relationships that could be construed as a potential conflict of interest.

## Publisher’s note

All claims expressed in this article are solely those of the authors and do not necessarily represent those of their affiliated organizations, or those of the publisher, the editors and the reviewers. Any product that may be evaluated in this article, or claim that may be made by its manufacturer, is not guaranteed or endorsed by the publisher.
